# 2108. In-vitro Activity of Five Novel Anti-Fungal Compounds Against Thirty-Two Strains of *Talaromyces marneffei*

**DOI:** 10.1093/ofid/ofad500.1732

**Published:** 2023-11-27

**Authors:** Joseph Barwatt, Thu Nguyen, Heera Natesan Sambath, Thuy Le

**Affiliations:** Duke University School of Medicine, Durham, North Carolina; Duke University School of Medicine, Durham, North Carolina; Duke University, Durham, North Carolina; Duke University School of Medicine, Durham, North Carolina

## Abstract

**Background:**

Talaromyces marneffei (Tm) is a thermally dimorphic fungus causing invasive mycosis in human immunodeficiency virus (HIV) infected patients in Southeast Asia and is a leading cause of AIDS-related deaths in this region with mortality on anti-fungal therapy as high as 30%. The limited options, routes of administration, and toxicity of antifungals with established efficacy against Tm calls for assessment of efficacy for additional antifungals against Tm. Here we evaluate the in-vitro efficacy of 5 novel anti-fungal agents against 32 clinical strains of Tm. The agents tested include ibrexafungerp, two manogepix compounds (APX001A and APX2039), olorofim, and oteseconazole.

**Methods:**

32 Tm isolates were cultured from clinical samples from patients in Vietnam and identified as Tm by standard morphological characteristics. Inocula were prepared from these clinical samples in conjunction with CLSI standards and plated onto 96-well microplates. Concentrations for each drug ranged from 0.008 µg/mL to 4 µg/mL. AlamarBlue^TM^, a resazurin-based cell viability indicator dye, was added to the drug-inoculum solution. Use of this reagent enabled utilization of a microplate fluorescence intensity(FI) reader to objectively identify the designated MIC endpoint of 50% inhibition of growth.

Methods (Figure 1). MIC endpoint determination using AlamarBlue indicator dye method.

**Figure:**
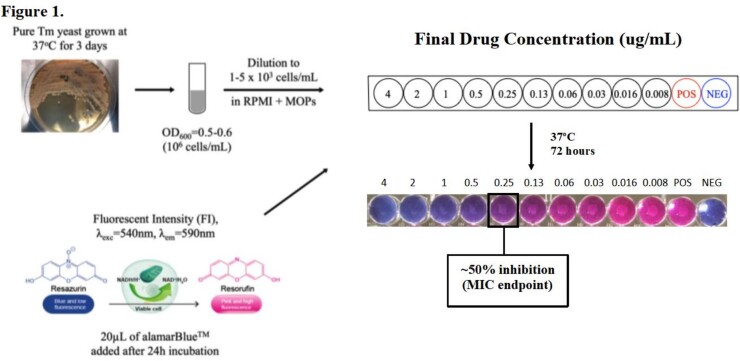

Clinical Tm isolates were thawed and plated on Yeast Peptone Dextrose (YPD) agar and grown for 3-5 days at 37°C. Isolates were then sub-cultured on fresh YPD agar and allowed to grow for 3 days prior to inoculum preparation. Inocula were prepared according to CLSI standards. Following suspension in solution, inocula were diluted in PBS until an optical density (OD) of 0.5 to 0.6 was achieved. Inocula were further diluted in RPMI + MOPS and added to the drug-dilution microplates to achieve a final concentration of 1000-5000 cells/mL. After incubation at 37°C for 24 hours, AlamarBlue was added to all wells. Following incubation for an additional 48 hours, fluorescence intensity (FI) was measured using a microplate reader to determine the MIC endpoint of 50% inhibition of fungal growth.

**Results:**

Of the drugs tested, olorofim showed the highest activity against the 32 isolates, with MICs consistently ≤ 0.008 µg/mL. This was followed by oteseconazole with MICs between ≤ 0.008 and 0.031 µg/mL APX001A with MICs between 0.031 and 0.25 µg/mL and APX2039 with MICs between 0.25 and 4 µg/mL. Ibrexafungerp had the broadest MIC distribution with MICs between 0.063 and 4 µg/mL.

**Figure:**
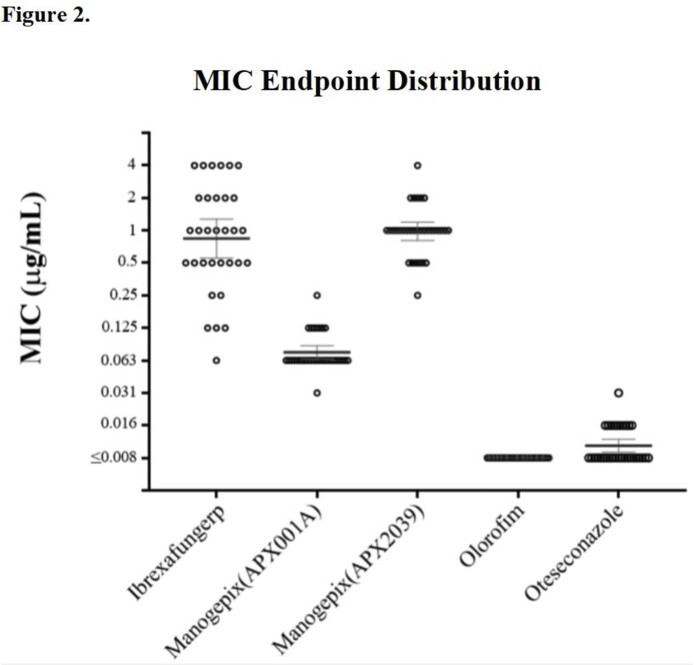

Figure 2/Table 1. MIC Endpoint Distribution.

**Figure:**
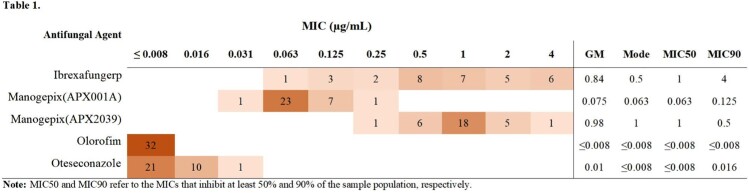

Figure 2 shows a scatter plot of MIC endpoints for each drug. Error bars demonstrate the geometric mean and 95% confidence intervals. Table 1 illustrates the MIC endpoint distribution in a graded heat map format. In addition, the absolute values of the geometric mean (GM), mode, MIC50, and MIC90 are displayed at the right side of the table. Both Figure 2 and Table 1 illustrate high efficacy for olorofim and oteseconazole, with low MIC endpoints consistent across the 32 isolates. The manogepix compounds(APX001A and APX2039) demonstrate surprisingly contrasted performance against Tm, despite the similar chemical structure and identical mechanism of action of the drugs. APX001A had significantly lower MIC endpoints compared to APX2039. Ibrexafungerp had the broadest range of MIC endpoints from 0.063 to 4µg/mL and the highest MIC50 and MIC90 of all the drugs. However, a significant proportion of isolates were highly susceptible to the drug.

**Conclusion:**

In-vitro activity of the 5 drugs tested against the 32 Tm strains was variable, but all should be considered potential candidates for treating Tm infection. To further evaluate the potential role of each drug in treating Tm infection, the next step should be to evaluate each in in-vivo models.

**Disclosures:**

**All Authors**: No reported disclosures

